# Recent advances in understanding the Th1/Th2 effector choice

**DOI:** 10.12703/r/10-30

**Published:** 2021-03-15

**Authors:** Matthew J Butcher, Jinfang Zhu

**Affiliations:** 1 Molecular and Cellular Immunoregulation Section, Laboratory of Immune System Biology, National Institute of Allergy and Infectious Diseases, National Institutes of Health, Bethesda, MD, USA

**Keywords:** Th1/Th2 effector choice, dendritic cells, innate lymphoid cells

## Abstract

For over 35 years since Mosmann and Coffman proposed the seminal “type 1 T helper (Th1)/type 2 T helper (Th2)” hypothesis in 1986, the immunological community has appreciated that naïve CD4 T cells need to make important decisions upon their activation, namely to differentiate towards a Th1, Th2, Th17 (interleukin-17-producing T helper), follicular T helper (Tfh), or regulatory T cell (Treg) fate to orchestrate a variety of adaptive immune responses. The major molecular underpinnings of the Th1/Th2 effector fate choice had been initially characterized using excellent reductionist *in vitro* culture systems, through which the transcription factors T-bet and GATA3 were identified as the master regulators for the differentiation of Th1 and Th2 cells, respectively. However, Th1/Th2 cell differentiation and their cellular heterogeneity are usually determined by a combinatorial expression of multiple transcription factors, particularly *in vivo*, where dendritic cell (DC) and innate lymphoid cell (ILC) subsets can also influence T helper lineage choices. In addition, inflammatory cytokines that are capable of inducing Th17 cell differentiation are also found to be induced during typical Th1- or Th2-related immune responses, resulting in an alternative differentiation pathway, transiting from a Th17 cell phenotype towards Th1 or Th2 cells. In this review, we will discuss the recent advances in the field, focusing on some new players in the transcriptional network, contributions of DCs and ILCs, and alternative differentiation pathways towards understanding the Th1/Th2 effector choice *in vivo*.

## Introduction

The ability of naïve CD4 T cells to differentiate into distinct cytokine-producing effector T helper (Th) cell subsets has been well appreciated over the last 35 years. The initial hypothesis set forth by Mosmann and Coffman in 1986 that at least two subsets of CD4 Th cell clonotypes could be distinguished based on the production of interferon (IFN)γ or IL-4^1^ has since been expanded to encompass new Th subsets, including type 1 IFNγ-producing Th (Th1) cells, type 2 IL-4/IL-5/IL-13-secreting Th (Th2) cells, IL-17A/IL-17F/IL-22-secreting Th (Th17) cells, T follicular helper (Tfh) cells, and regulatory T cell (Treg) populations^2^. Indeed, the initial hypothesis set forth sparked a period of discovery in which the major molecular and cellular events leading up to the differentiation of naïve CD4 T cells towards Th1 and Th2 effector cells were characterized using excellent reductionist *in vitro* models.

Th1 cells are key players in helping to mount a host defense against intracellular pathogens, including protozoa, bacteria, and viruses, but are also involved in the development of certain types of autoimmune diseases^3–5^. Lineage-specific master transcription factors often play decisive roles in determining cell fate. Following Mosmann and Coffman’s hypothesis, T-bet was identified^6–8^ as the Th1-lineage master transcription factor, as T-bet directly regulates the production of IFNγ. Soon after, several distinct upstream regulatory pathways were described to promote Th1 cell differentiation. As T-bet can positively regulate IFNγ production, autocrine IFNγ–IFNγR–Stat1 signaling can reinforce T-bet expression to solidify the Th1 phenotype^9,10^. IL-12 can also potently induce T-bet expression and Th1 polarization independent of IFNγ signaling^11,12^. Additionally, at the onset of an infection, IL-27 can induce IL-12R on naïve CD4 T cells, making them more susceptible to IL-12-mediated T-bet expression and Th1 polarization^13^. Lastly, T-bet was reported to induce its own expression^14^. However, T-bet autoregulation may not be required in the presence of either IL-12 or IFNγ. Nevertheless, T-bet and IL-12-induced pStat4 may synergize to remodel the *Ifng* locus and optimally induce IFNγ production^12^.

In contrast to Th1 cells, Th2 cells are primarily important in helping to mount a defense against helminth infections and exposure to venoms, but they also participate in different types of allergic diseases including asthma, atopic dermatitis, allergic rhinitis, and food allergy^15–19^. Ten years after the Th1/Th2 hypothesis, GATA3 was identified as the master transcription factor responsible for Th2 cell differentiation^20–23^. However, unlike T-bet, which is induced during Th1 cell differentiation, GATA3 is already expressed by naïve CD4 T cells at low levels and is required for CD4 T cell development in the thymus^24,25^. Upon encountering antigen presentation and IL-4, activation of Stat6 is sufficient to induce GATA3 upregulation and Th2 polarization. However, GATA3 is also sensitive to the strength of T cell receptor (TCR) stimulation, as low-dose/weaker TCR stimulation is sufficient to upregulate GATA3 expression in the absence of IL-4/Stat6 signaling^26^, consistent with the notion that TCR signaling strength could affect the fate of T cell differentiation^27–29^. Thus, there are IL-4-dependent and IL-4-independent mechanisms of GATA3 induction and Th2 cell differentiation, particularly *in vivo*, and GATA3 is critical for Th2 cell differentiation both *in vitro* and *in vivo*^22^. GATA3 directly binds to the *Il4/Il13* gene locus. While GATA3 can induce *Il5* and *Il13* transcription through binding to their promoters^30,31^, GATA3 mainly affects *Il4* expression through regulating epigenetic modifications at the Th2 cytokine gene locus^25^.

Following the identification of T-bet and GATA3 as Th1- and Th2-polarizing transcription factors, respectively, it became readily apparent that lineage cross-regulation occurs in order to solidify one T effector fate over the other. For example, T-bet was shown to suppress GATA3 transcription^12,32^ and inhibit GATA3 function through direct protein–protein interaction^33^. In addition, T-bet and GATA3 both colocalize at key Th1- and Th2-related genes, and endogenous T-bet is sufficient to inhibit GATA3 function during Th1 polarization, thereby enforcing a Th1 program^12,34,35^. In contrast, during Th2 polarization, GATA3 may suppress Stat4 expression, suppress Runx3-mediated IFNγ production, and epigenetically silence the *Tbx21* locus to ensure Th2 polarization^25,36,37^.

In this review, we will discuss some recent interesting advances towards understanding the Th1/Th2 effector cell “choice”, particularly during *in vivo* immune responses, which include the role of new players in the transcriptional network, the contributions of dendritic cells (DCs) and innate lymphoid cells (ILCs) in the initiation of T cell differentiation, and the alternative differentiation pathways transiting from Th17 cells to Th1 or Th2 cells. While some of the topics that will be discussed are also relevant to Th17-, Treg-, and Tfh-mediated cellular responses as well as their plasticity, these subsets will not be discussed in detail, and we refer the reader to several excellent reviews^2,38–45^.

## New roles for known transcription factors in regulating the differentiation and functions of Th1 and Th2 cells

Despite all that we have learned about the Th1/Th2 dichotomy in the past 35 years, there is still much to learn about the Th1/Th2 choice in the context of transcriptional networks. Specifically, non-lineage-specifying transcription factor networks can influence the quality of a Th1 or Th2 response by influencing their cytokine repertoire. Interestingly, several recent studies have highlighted non-lineage-restricted transcription factors, Bhlhe40 and B cell lymphoma 11B (Bcl11b), in affecting the cytokine repertoires of Th1 and Th2 cells.

Three reports have recently shown Bhlhe40 to be a key non-lineage-related cytokine modulator, demonstrating a role for Bhlhe40 in Th1 immunity in *Toxoplasma gondii* and *Mycobacterium tuberculosis* infection models and in Th2 immunity in a model of *Heligmosomoides polygyrus* infection^46–48^. Two groups independently demonstrated that Bhlhe40 plays a key role in suppressing IL-10 production by Th1 cells, functioning as a key inflammation/anti-inflammation switch. Yu and colleagues^46^ demonstrated that a CD4 T cell-restricted knockout of Bhlhe40 resulted in increased IL-10 production and decreased IFNγ production by T cells in a *T. gondii* infection model. Bhlhe40 may suppress IL-10 production via suppression of c-Maf and/or Aiolos but promotes IFNγ production in a T-bet-independent manner. Similarly, Huynh and colleagues^47^ demonstrated that Bhlhe40 is an essential repressor of IL-10 during *M. tuberculosis* infection. In the context of *H. polygyrus* helminth infection, Jarjour and colleagues^48^ found that Th2 cells require Bhlhe40 in order to mount an effective anti-helminth immune response. Interestingly, in their model, Bhlhe40 controlled the production of granulocyte-macrophage colony-stimulating factor (GM-CSF) and IL-5 cytokines within gut Th2 cells, and both cytokines are required for efficient eosinophil recruitment and helminth control, suggesting that Bhlhe40 plays a key role in controlling the production of GM-CSF, IL-10, and other cytokines in multiple T cell subsets, including Th1, Th17, and Th2 cells. All together, these data suggest that Bhlhe40 plays an important lineage-independent cytokine-modifying role in Th1, Th2, and Th17 cells by promoting inflammation via inducing GM-CSF and suppressing IL-10.

There have also been several recent reports that have highlighted a non-lineage modulatory role for Bcl11b on Th1 and Th2 responses. Bcl11b is a critical transcription factor for early T cell development and is expressed by all T cells starting from the CD4/CD8 double negative (DN) stage 2^49^. Bcl11b is critically required for Vβ-DJβ recombination and *Tcrb* expression at the DN3 to DN4 transition, as well as positive selection at the CD4^+^CD8^+^ DP stage^50,51^. In addition, Bcl11b plays an important role in regulating the development and functions of mature T cell subsets^52^. Furthermore, Bcl11b suppresses the cell fate of natural killer cells and is important for the development of ILC2s^53–57^. Recently, Fang and colleagues^58^ have demonstrated a novel role for Bcl11b in suppressing Th1 cell differentiation while simultaneously limiting the expression of Th2 cell-associated genes. In this study, it has been shown that Bcl11b physically interacts with GATA3 through protein–protein interaction and binds to common cis-regulatory elements of lineage-related genes that GATA3 binds in Th2 cells and thus limits IL-4, IL-5, and IL-13 production both *in vitro* and *in vivo*. Interestingly, GATA3 and Bcl11b also simultaneously suppressed Th1-associated genes by modulating H3K27ac and DNase I hypersensitivity sites within these gene loci. Strikingly, while Bcl11b limits Th2 cell responses at a later stage, it plays an important role in the initiation of Th2 responses^59^. Deletion of Bcl11b in naïve CD4 T cells results in a reduced Th2 response during helminth infection and in allergic asthma models, presumably because of a dysregulated balance between GATA3 and Runx3 expression in the absence of Bcl11b. Interestingly, Bcl11b may also play a role in restricting the expression of Th2 lineage genes within Th17 cells, as Bcl11b-deficient Th17 cells were shown to express GΑΤΑ3, IL-4, α4β7, and CCR9 in an experimental autoimmune encephalomyelitis (EAE) model^60^. Therefore, Bcl11b is not only critical for T cell development in the thymus but also important for T cell differentiation in the periphery, and its functions are highly cell type (or developmental stage) specific.

Interestingly, the aforementioned effects of Bhlhe40 and Bcl11b on the activation and differentiation of Th2 cells were recently confirmed by Henriksson and colleagues^61^. Expanding on the previous work focused on the network of transcription factors involved with Th17 cell activation versus differentiation^62^, Henriksson and colleagues utilized a combination of genome-wide CRISPR knockout libraries combined with RNAseq, ATAC-Seq, and ChIP-seq to dissect out the regulatory circuitry controlling the activation versus differentiation of Th2 cells *in vitro*. As a result of their efforts, they not only confirmed GATA3, Stat6, Batf, PPARγ, and IRF4 as key transcription factors that are involved in Th2 cell differentiation and activation but also revealed Bhlhe40, Bcl11b, and Xbp1 as transcription factors that are involved in Th2 cell activation. While more factors that are involved in regulating Th1/Th2 differentiation and functions are still being discovered, including the p53 family protein p73, which affects Th1 cell differentiation^63^, and Blimp-1, which regulates GATA3 expression and thus Th2 cell differentiation^64^, the combination of novel genome-level technologies such as CRISPR knockout with high-throughput sequencing will hopefully reveal more non-lineage-related transcription factors in modulating CD4 T cell differentiation.

## DC subsets in making the Th1/Th2 decision

**Figure 1.**
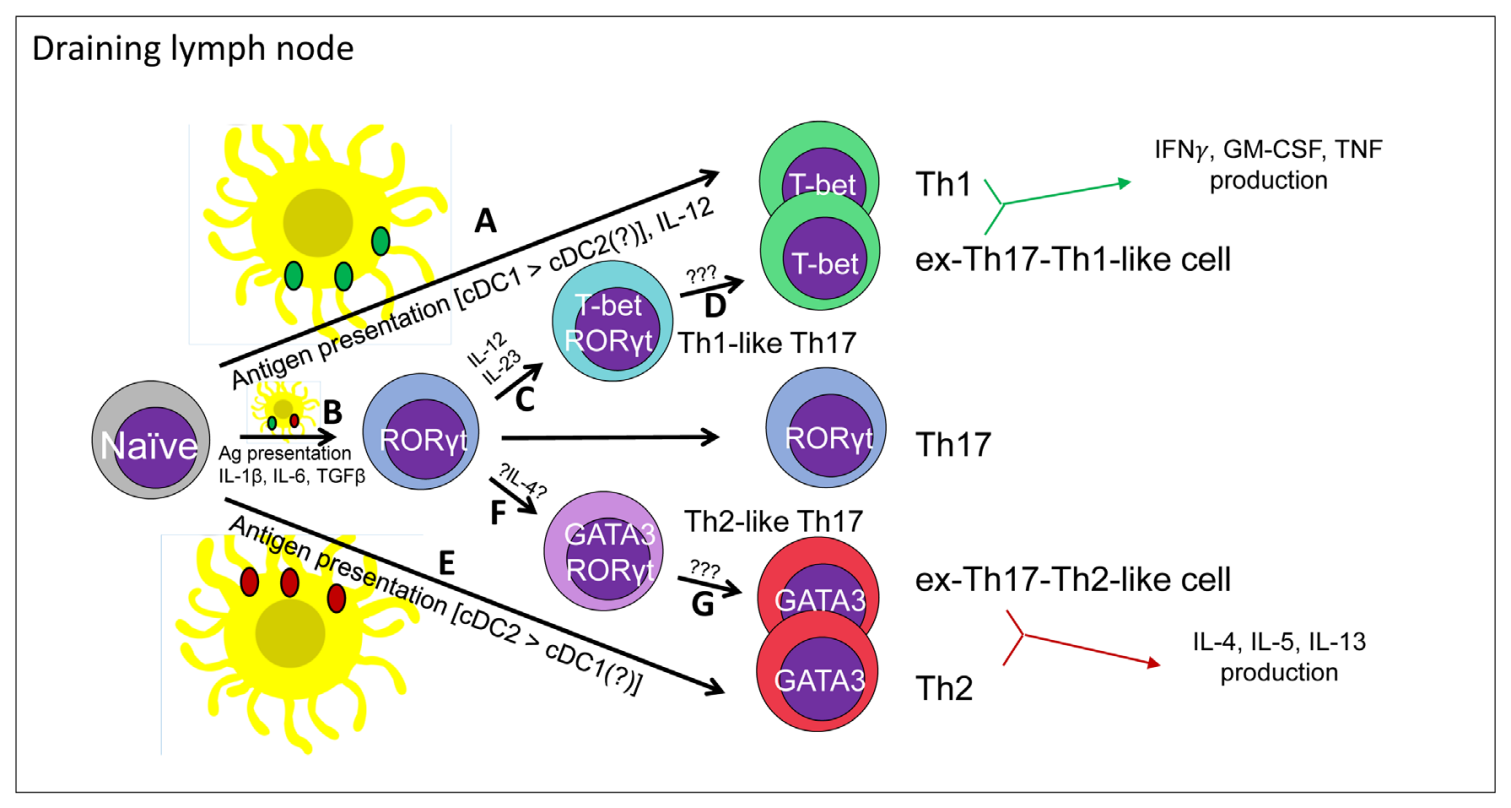
Contributions of dendritic cell subsets in the initiation of classical and alternative differentiation pathways for the generation of Th1 and Th2 cells *in vivo*. Presented here is our updated view of the Th1/Th2 T effector decision *in vivo*. In response to an *in situ* immunological insult, local antigen-presenting dendritic cells acquire Ag and home to the nearest draining lymph node to present Ags. In the case of a pro-Th1 insult, such as a bacterial infection, bacterial Ag-laden dendritic cells can present bacterial Ags to naïve T cells and produce IL-12 in order to help generate bacterial Ag-specific Th1 cells (**A**). In parallel, some bacterial PAMPs can trigger Ag-laden dendritic cells to produce pro-Th17 cytokines, including IL-1β, IL-6, and TGFβ (**B**). Bacterial Ag-specific Th17 cells can subsequently respond to IL-12 and/or IL-23 in order to generate T-bet^+^ Th1-like Th17 cells (**C**) and ex-Th17-Th1-like cells (**D**) via unclear mechanisms. In contrast to a pro-Th1 insult, allergen exposure or a helminth infection is able to elicit a Th2 Ag-specific T cell response. In this scenario, helminth- or allergen-Ag-bearing dendritic cells home to the nearest draining lymph node, where they may select for Ag-specific naïve T cells to generate an Ag-specific Th2 cell response (**E**). In parallel, some helminth- or allergen-Ag-laden dendritic cells may instead help to generate Th17 cells, which may subsequently give rise to GATA3^+^ Th2-like Th17 cells (**F**) and ex-Th17-Th2-like cells (**G**). Ag, antigen; cDC, conventional dendritic cell; GM-CSF, granulocyte-macrophage colony-stimulating factor; IFN, interferon; IL, interleukin; PAMP, pathogen-associated molecular pattern; TGFβ, transforming growth factor beta; Th1, type 1 T helper; Th2, type 2 T helper; Th17, interleukin-17-producing T helper; TNF, tumor necrosis factor.

In order for naïve T cells to differentiate towards Th1 or Th2 effector cell fates *in vivo*, TCR stimulation via antigen presentation, co-stimulation, and polarizing cytokine cues, such as IL-12, IL-27, etc., are required. DCs are the premier antigen-presentation cell population *in vivo*, and they are ultimately required to activate and expand antigen-specific CD4 T cells via peptide–MHCII–TCR interactions (Figure 1). As such, DCs and DC subsets have garnered attention in the literature based on their differences in antigen presentation and T cell polarization capabilities. While the topic of DC subsets has been expertly reviewed elsewhere^43,65,66^, briefly, there are several subsets of DCs: conventional type 1 DCs (cDC1s), conventional type 2 DCs (cDC2s), plasmacytoid DCs (pDCs), monocyte-derived DCs (moDCs), and Langerhans cells (LCs), which differ based on their anatomical locations, ontogeny, and antigen-presentation capacities. Interestingly, out of the aforementioned subsets, conventional DCs are the most abundant and have been demonstrated to differ in terms of their abilities to induce Th1 or Th2 responses.

To efficiently polarize a naïve T cell to a Th1 phenotype, pro-Th1 polarizing cytokines are required, such as IL-12 and IL-27. Interestingly, cDC1s are a major source of IL-12 *in vivo*^67–69^ and have been reported to be superior in terms of their ability to generate Th1 cells in *ex vivo* coculture systems^70^ when they are compared to cDC2s. cDC1s constitutively express *Il12b* transcript and produce IL-12p40 protein *in vivo*^71,72^. Additionally, in experimental models of Th1 inflammation in the absence of cDC1s, the Th1 response is significantly compromised^69,73–77^, suggesting that cDC1s play a major role in generating Th1 responses *in vivo*. However, the following question arises: why are cDC1s able to produce basal levels of IL-12? The constitutive production of IL-12p40 doesn’t seem to depend on commensals or the specific acquisition of antigen, as cDC1 IL-12p40 production is maintained in germ-free, naïve mice, suggesting that IL-12 production is either an intrinsic property of the cDC1 lineage or maintained by the microenvironment^78^. Homeostatic cDC1-derived IL-12 might function to support the generation of innate-like T-bet^high^ CD4 memory phenotype cells. Interestingly cDC1s aren’t the only DC subset that is able to initiate a Th1 response. There is some evidence to suggest that moDCs are also capable of driving Th1 responses during *T. gondii* and *Salmonella* infections as well as in immunizations with CpG or CFA-based adjuvants^79–82^, possibly in coordination with cDC1s. Furthermore, during inflammation, cDC2s may acquire a hybrid inflammatory cDC2 phenotype in a manner that is reminiscent of cDC1s and moDCs, and type 1 IFN drives the generation of inflammatory cDC2s, which are capable of priming naive CD4 T cells to become IFNγ-producing Th1 cells^83^. Another recent study has also demonstrated that TNFR2^+^ cDC2 cells are able to drive Th1 responses following an intranasal immunization with cyclic dinucleotide as an adjuvant^84^, suggesting that both the cDC subsets and the adjuvant/PAMPs involved are important in determining the T cell differentiation outcome. Lastly, as one might expect, TLR3 and TLR9 agonists enhance DC IL-12p40 production and thus Th1 cell differentiation.

In contrast to the role of cDC1s in generating and recruiting Th1 cells, cDC2s, including IRF4^+^ cDC2s (some of which also express the transcription factor Klf4) in the skin, lungs, and intestinal lamina propria, are necessary for triggering Th2 responses in models of helminth infection or allergic diseases^76,85–88^. The exposure of cDC2s to helminth products, like *Schistosoma mansoni* egg antigen (SEA)-derived protein Omega1, can endow cDC2s with the capacity to induce Th2 cell differentiation by inhibiting IL-12 production and limiting contact time with CD4 T cells, resulting in Th2-favorable antigen presentation conditions^88–94^. Interestingly, transcriptomic analyses of helminth or allergen-conditioned DCs have identified TSLP as a key upstream pathway involved in the upregulation of pro-Th2 OX40L expression. CD301b^+^ dermal DCs can also support Th2 cell differentiation, as an immunization with OVA mixed with papain or alum is sufficient to drive Th2 cell polarization^95^. Interestingly, allergen-activated TRPV1 neurons may trigger the migration of CD301b^+^ DCs to the draining lymph node via substance P to induce Th2 cell differentiation^96^. Thus, a combination of select DC subsets, pro-Th1 or pro-Th2 adjuvants, PAMPs, and the site(s) of antigen acquisition seem to ultimately determine the resulting Th1 or Th2 response *in vivo* rather than a T cell “choice” within the draining lymph node.

## The involvement of local ILC populations on determining T cell differentiation

ILCs are innate lymphocytes that lack specific antigen receptors but are able to respond to alarmin cytokines in order to closely mirror T cell subsets in terms of their subsets and cytokine repertoires. As a result, ILCs have drawn interest in how they might regulate the initiation or quality of a Th1/Th2 response *in situ*. As ILCs have been expertly reviewed elsewhere^97–99^, we will briefly re-introduce them here. ILCs can be divided up into five major subsets: NK cells, which mirror CD8 T cells, express T-bet and Eomes, and produce IFNγ, Perforin, and Granzyme B; group 1 ILCs (ILC1s), which closely mirror Th1 cells, express T-bet, and produce IFNγ and TNF; group 2 ILCs (ILC2s), which closely mirror Th2 cells, express high levels of GATA3, and produce IL-5, IL-13, and IL-9; group 3 ILCs (ILC3s), which closely mirror Th17 cells, express RORγt (with some of them also expressing T-bet), and produce IL-22 and GM-CSF (also IFNγ for T-bet^+^ ILC3s); and lymphoid tissue inducer (LTi) cells, which express RORγt, produce RANK, lymphotoxins, TNF, and IL-17A, and are required for the formation of lymphoid tissues during development. Thus, as one might expect, ILC subsets are active participants during immune responses and have garnered interest in how they might influence the development of Th1 or Th2 immune responses.

### The effects of ILC-derived cytokines on Th responses

**Figure 2.**
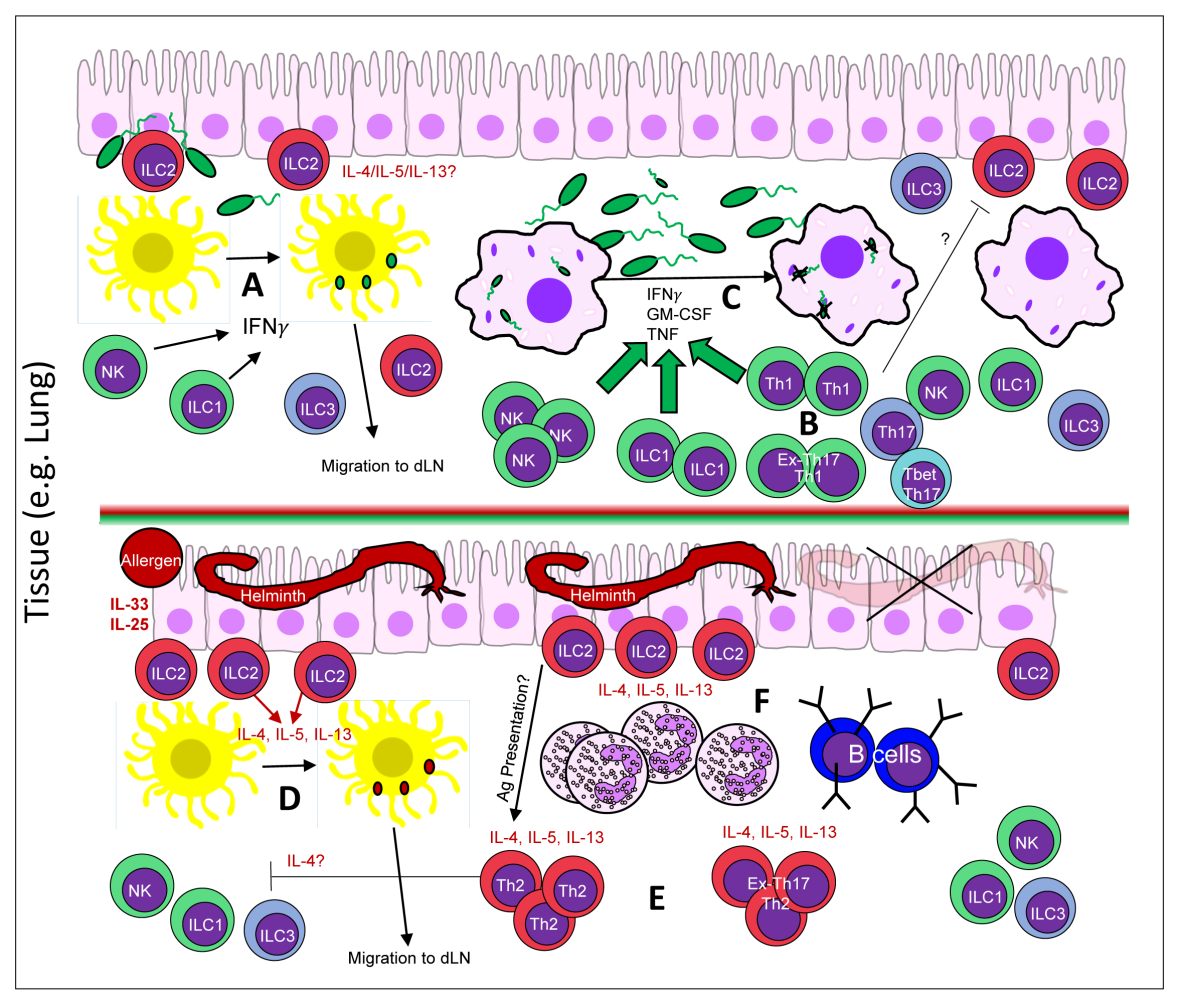
An updated view of the players *in situ* that help to shape a Th1/Th2 response. Presented here is our updated view of the Th1/Th2 T effector decision *in situ*/*in vivo*. In response to an immunological insult in a complex tissue, such as the lung, local innate immune cells respond appropriately to guide the downstream selection of Th1 or Th2 cells. In response to a pro-Th1 bacterial infection (**A**–**C**), *in situ* NK cells and ILC1s respond to alarmins to produce IFNγ and local dendritic cells acquire bacterial Ags (**A**). Ag-laden dendritic cells may then travel to the nearest dLN to present bacterial Ags and generate bacterial Ag-specific Th1 cell or a mixed Th17 and Th1 cell response. Bacterial Ag-specific Th1, Th17, T-bet^+^ Th1-like Th17, and ex-Th17-Th1-like cells can then home back to the site of infection or Th17 cells can generate T-bet^+^ Th1-like Th17/ex-Th17-Th1-like cells *in situ* (**B**) in order to coordinate with local NK cells, ILC1 cells, and macrophages to control the bacterial infection (**C**). The potential antagonistic effects between Th1 cells and lung-resident ILC2s in this context are unclear. In contrast to a pro-Th1 insult, allergen exposure or a helminth infection is able to elicit a Th2 Ag-specific cell response (**D**–**F**). In this scenario, a helminth infection is sufficient to drive the production of alarmins (IL-33/IL-25) from the lung epithelium and activate local tissue ILC2s (**D**). *In situ* ILC2s can produce IL-4/IL-13 in response and promote helminth Ag-bearing dendritic cells homing to the nearest dLN. Helminth Ag-specific naïve T cells are selected by the Ag-laden dendritic cell and helminth Ag-specific Th2 or a mixture of Th17 and Th2 cells are generated (**E**). Helminth Ag-specific Th2, Th17, Th2-like Th17 cells, and ex-Th17-Th2-like cells may then home back to the infected tissue in order to work in conjunction with locally recruited and activated eosinophils, B cells, and ILC2s in order to expel or kill the invading helminth (**F**). Ag, antigen; dLN, draining lymph node; GM-CSF, granulocyte-macrophage colony-stimulating factor; IFN, interferon; IL, interleukin; ILC, innate lymphoid cell; NK, natural killer; Th2, type 2 T helper; Th17, interleukin-17-producing T helper; TNF, tumor necrosis factor.

So how might ILCs influence the generation of a T cell response? One obvious way would be via the secretion of pro-Th1 or pro-Th2 cytokines in response to tissue alarmins (Figure 2). As one might expect, type 2 immunity-inducing agents (e.g. helminth products, papain, Der p1, etc.) can disrupt the integrity of the epithelium, resulting in the release of alarmins, including IL-33, IL-25, and TSLP. ILC2s can respond to IL-33 via T1/ST2 (IL-33R) and IL-25 via IL-17RB to locally produce the type 2 cytokines IL-4, IL-5, and IL-13, thereby setting up a pro-Th2 milieu *in situ* and helping to initiate and maintain a Th2 response^17,100^. With respect to IL-4, while ILC2s are relatively poor IL-4 producers compared to Th2 cells, there is some evidence to suggest that ILC2-derived IL-4 may support Th2 cell differentiation. During *H. polygyrus* infection, it seems that ILC2-derived IL-4 plays an important role in inducing a Th2 response^101^. In addition, it has been shown that other inflammatory mediators, such as the leukotriene LTD4, may induce IL-4 production by ILC2s^101–103^. Furthermore, IL-13 produced by ILC2s may promote the migration of lung DCs into the draining lymph node to initiate Th2 responses^104^. Similarly, *in situ* production of IL-4 and IL-13 by ILC2s or Th2 cells may further induce the expression of IL-25 to amplify type 2 immune responses through recruiting more activated ILC2s^105^. Lastly, early production of IL-5 by *in situ* ILC2s can potentially support a Th2 response via the recruitment of IL-4-producing eosinophils^106–110^. Therefore, the crosstalk between ILC2s and Th2 cells may play an important role in mounting a robust type 2 response, and such crosstalk may also serve as a target for treating chronic type 2 inflammation.

In response to an acute pro-Th1 infectious insult, such as *T*. *gondii* or MCMV infection, ILC1s are stimulated by cDC-sourced IL-12 in order to produce IFNγ and can help mount a Th1 response. However, one open-ended question considering this paradigm concerns how ILCs and DCs might interact or crosstalk in order to influence the generation of a Th1 or Th2 response. As noted in the preceding section, while cDC1s are great at generating a Th1 response, given the right adjuvants TNFR2^+^ cDC2s can also elicit Th1 cells. Depending on the adjuvants and infection route/immunization route involved, the involvement of ILC1s versus ILC2s might differ, as the tissue distributions of both are quite different. Another open-ended question that has not been addressed in the literature concerns how opposed ILCs might respond during a Th1 or Th2 response. For example, the lung is host to a large population of ILC2s and a smaller population of ILC3s and ILC1s, and, as one might expect, ILC2s actively participate in Th2-mediated lung pathologies including allergy. However, what might happen to those ILC2s in the context of a strong Th1 infectious insult such as a viral infection or bacterial pneumonia? Is it possible that the activation status of ILC2s in the lung may determine the severity of SARS-CoV2-infected patients? Thus, while ILCs are active participants in setting up a local pro-Th1 or pro-Th2 environment, there is still much to learn.

### The effects of ILC-mediated antigen presentation on Th responses

Another possible mechanism through which ILCs might affect the Th1/Th2 response is through potential antigen presentation. Unlike MHC-I, which is expressed by almost every cell, the expression of MHC-II is restricted to antigen-presenting cells, and, remarkably enough, some ILC2s and ILC3s are also endowed with the machinery to process and present peptides on MHC-II molecules and are thus potentially able to interact with T cells via TCR–peptide–MHCII complexes^99,111,112^. Interestingly, ILC2s can stimulate T cells via peptide-loaded MHC-II, and one report demonstrated that the secretion of IL-2 by ILC2s in T cell/ILC2 co-cultures resulted in an expansion of ILC2s^112^. Thus, ILC2s might also be able to influence *in situ* Th2 responses via antigen presentation. At present, it isn’t clear how important this mechanism is in comparison to antigen presentation via professional antigen-presenting DCs.

## Alternative differentiation pathways to Th1 & Th2 cells via Th17 intermediates

In addition to Th1 and Th2 cells, a third type of CD4 effector T cell termed Th17 cells are interesting in the context of the Th1/Th2 fate decision owing to their less-committed, plastic properties. Briefly, Th17 cells are a bona fide IL-17A/IL-17F/IL-22-secreting CD4 T effector cell subset that plays a key role in the defense against opportunistic fungal or bacterial pathogens but may also participate in autoimmune and allergic diseases^38^. Following the discovery of IL-17A-producing CD4 T cells in 2005^113,114^, the mechanisms of Th17 polarization were quickly described^115–118^. Conceptually, extracellular bacterial or fungal PAMPs result in antigen-presenting cell-mediated antigen presentation and the production of the pro-inflammatory cytokines IL-1β, IL-6, and IL-23, which drive the generation of Th17 cells in a RORγt (TCR/NFAT/NFkB/AP-1) and pSTAT3 (IL-6 and IL-23) dependent manner^38,119^. In addition, the master Th17 lineage transcription factor RORγt can collaborate with other transcription factors, including IRF4, BATF, and Runx1/Runx3, to optimally induce the expression of Th17 lineage genes^2,62,120^. However, following these seminal discoveries, it became readily apparent that Th17 cell-mediated responses can exert both protective and pathogenic effects during immunological challenges, suggesting that Th17 cells can be divided into “pathogenic” and “non-pathogenic” subsets. Functionally distinct homeostatic and inflammatory Th17 cells can be found in the intestine^121^. Several putative regulators of Th17 “pathogenicity” have been described, including IL-23, CD5L, REV-ERBα, and various environmental factors such as commensal organisms and tissue salinity; however, the precise mechanisms governing the protective/pathogenic switch are still unclear and may involve supplemental non-lineage-related transcription factors^122–128^. To further complicate things, it has become readily apparent that Th17 cells can co-opt T-bet or GATA3 expression to assume aspects of the Th1 or Th2 lineage (termed Th17/Th1 or Th17/Th2 cells) or to fully assume a functional Th1 or Th2 phenotype (ex-Th17-Th1-like cell or ex-Th17-Th2-like cell).

### When the distinctions between Th17 and Th1 cells blur: Th17/Th1 and ex-Th17-Th1-like cells

To date, there are several lines of evidence to support the concept that Th17 cells may co-opt aspects of the Th1 lineage or even assume an ex-Th17-Th1-like cell fate in various *in vivo* contexts. In a study utilizing IL-17A^+^ Th17 cell fate mapping reporter mice (*Il17a*^Cre^*Rosa26*^eYFP^ mice) and mouse models of Th17- and Th1-related inflammation (EAE and subcutaneous immunization with *Candida albicans*), a population of fate mapped ex-Th17 cells (IL-17A^–^eYFP^+^ cells) was identified^129^. Interestingly, subpopulations of T-bet^+^RORγt^+^IL-17A^+^ cells and IFNγ^+^IL-17A^–^ arose amongst the IL-17A fate mapped cells, suggesting that a Th17 cell may generate a mixture of IFNγ^+^IL-17A^+^ Th17 cells and IFNγ^+^IL-17A^–^ ex-Th17-Th1-like cell subpopulations. Furthermore, the generation of IL-17A fate mapped eYFP^+^ cells depends on IL-23-induced expression of T-bet, as an *Il17a*^Cre^*Rosa26*^eYFP^*Il23r*^–/–^ variant failed to generate IFNγ^+^IL-17A^+^eYFP^+^ or IFNγ^+^IL-17A^–^eYFP^+^ fate mapped cells. Runx1 together with T-bet plays an important role in the generation of IFNγ-producing Th17 cells^130^. A similar phenotypic change from Th17 to Th1 cells has also been observed in a model of *Helicobacter hepaticus*-induced colitis, an *in vitro* polarized Th17 T cell transfer *Rag2*^–/–^ colitis model, and an IL-22 Th17 fate mapping model in the gut, suggesting that Th17 cells are intrinsically plastic and can generate a population of T-bet^+^RORγt^+^ Th1-like Th17 cells and a population of ex-Th17 cells that assumed a Th1 phenotype (ex-Th17-Th1-like cells)^131–133^. In addition, IL-23 was shown by Jain and colleagues to induce Blimp-1 expression within Th17 cells, and Blimp-1 was shown to be necessary for the induction of T-bet-, GM-CSF-, and IFNγ-expressing Th17 cells within the gut, suggesting that IL-23 along with other transcription factors may synergize to induce a Th1-like phenotype within Th17 cells^134^. However, from a Th1/Th2-centric viewpoint, the later ex-Th17-Th1-like cell population poses an interesting philosophical dilemma: if Th17 cells can generate a subpopulation of cells that are essentially Th1 cells and naïve T cells can directly differentiate into Th1 cells as well, what, if anything, would distinguish the two *in vivo*? In addition, are there any meaningful differences between former Th17-derived Th1 cells and *de novo* Th1 cells? Do they have distinct functionalities in host defense versus in inflammation? While these open questions remain to be addressed in the literature, Th1-like subpopulations of Th17 cells have been observed in various human patient populations, including multiple sclerosis, rheumatoid arthritis, psoriasis, inflammatory bowel disease, and *M. tuberculosis* patients and others, so Th1-like Th17 cells and ex-Th17-Th1-like cells may have some clinical relevance.

### Th17 plasticity towards the other fate: Th17/Th2 and ex-Th17-Th2-like cells

Similarly, there is some limited evidence to suggest that Th17 cells may also assume Th2-like properties in the context of allergic disease. In a study profiling human PBMC CD4 memory cells from atopic asthma patients, Wang and colleagues observed a population of CD4^+^CRTH2^+^CCR6^+^ T cells that were elevated in allergic asthmatic patients versus healthy controls and co-expressed IL-4, IL-17A, IL-22, IL-5, IL-13, RORγt, and GATA3, suggesting that a population of Th2-like Th17 cells are generated during the development of atopic asthma and may be associated with the pathology. Interestingly, a similar population of IL-4^GFP+^IL-17A^+^ Th17 cells were isolated from the bronchoalveolar lavage (BAL) fluid and lungs of *Aspergillus Orazae* + OVA challenged IL-4-GFP knock-in (4Get) mice, suggesting that both mouse and human Th17 cells may assume a partial Th2 phenotype in the context of allergy^135^. Similar results were observed by Irvin and colleagues in a study examining BAL T cell phenotypes from a different cohort of atopic asthmatic patients. In that study, IL-4^+^IL-17A^+^GATA3^+^RORγt^+^ T cells were observed and IL-4^+^IL-17A^+^ T cells correlated with eosinophil counts and occurred in the most severe subgroup of asthmatic patients^136^. Together, these observations of Th2-like Th17 cells suggest that Th17 cells may also assume characteristics of Th2 cells and suggest that the formation of ex-Th17-Th2-like cells (the Th2 equivalent of ex-Th17-Th1-like cells) may be theoretically feasible in the context of allergy or helminth infections. Indeed, a recent study showed that ~10% of the IL-17A^–^IL-4/5/13^+^ cells in the lung are IL-17A fate mapping positive^137^. Taken together, the available data indicate that close encounters between bona fide Th1/Th2 cells and Th17 plastic approximations of Th1/Th2 cells, which develop through the Th17 intermediate stage, may occur *in vivo*. While it is likely that the pro-inflammatory cytokines such as IL-1β and IL-6, both of which can be induced even during Th1 and Th2 responses, may determine these alternative differentiation pathways to Th1 and Th2 cells, the precise regulation and contributions of these unconventional Th1/Th2 subsets relative to classical Th1/Th2 cells during immune responses remain an open question (Figure 1).

## Conclusion

In summary, despite the tremendous amount of work that has been accomplished since Mossman and Coffman’s seminal Th1/Th2 hypothesis in 1986, there is clearly more to discover about how Th1/Th2 effector fate decisions are made *in vivo*. While the present work was in no way designed to be all encompassing, we have highlighted some recent advances in the field that have contributed to our understanding of how Th1/Th2 immune responses are initiated and amplified *in vivo*. Ultimately, the decision to launch or modify the quality of a Th1 or Th2 immune response can be distilled down to several variables: 1) the site(s) of antigen encounter and which DC subset(s) acquire and present the antigen(s), 2) which ILC subsets are activated *in situ* to support the *de novo* adaptive immune response, 3) which differentiating cytokines are produced by the antigen-presenting DCs within the draining lymph node, 4) whether or not there is Th17 cell involvement to contribute towards the overall Th1/Th2 cell response, and 5) the expression of Th1/Th2 response-modifying transcription factors, such as Bhlhe40 and Bcl11b, etc. New technologies including single cell RNA-Seq^138^ and single cell ATAC-Seq analysis of antigen-specific CD4 T cells as well as advanced imaging to visualize cell–cell interactions *in vivo* at different stages during immune responses will greatly help further our understanding of the differentiation process of Th1 and Th2 cells and ultimately contribute to the design of better and precise strategies in treating immunological diseases involving these two important lymphocyte subsets.
